# Physical activity of German children during different segments of the school day

**DOI:** 10.1007/s10389-016-0755-2

**Published:** 2016-08-03

**Authors:** Susanne Kobel, Sarah Kettner, Christine Lämmle, Jürgen M. Steinacker

**Affiliations:** Division of Sports and Rehabilitation Medicine, University Hospital Ulm, Frauensteige 6, Haus 58/33, 89075 Ulm, Germany

**Keywords:** Moderate to vigorous physical activity (MVPA), Physical education, Break times, Recess, Primary school children

## Abstract

**Aim:**

This study objectively investigated the amount and intensity of German primary school children’s physical activity (PA) during different segments of the school day and explored the contribution of physical education (PE) and break times to daily moderate to vigorous PA (MVPA).

**Subject and methods:**

PA of 294 children (7.1 ± 0.7 years, 48 % male) was objectively measured for 6 days using Actiheart®. Based on children’s timetables, break times and PE periods were determined and PA was calculated individually and subsequently classified in light (1.5–3 MET), moderate (3–6 MET) and vigorous (>6 MET) intensities. Weight status was determined during a school visit.

**Results:**

Children spent 133 ± 61 min in MVPA; on weekdays, this amount increased significantly (141 ± 66 min, *p* ≤ 0.01). 45.9 % of children reached physical activity guidelines of 60 min of MVPA daily, with boys achieving this goal significantly more often than girls (65.6 vs. 28.7 %, respectively; *p* ≤ 0.01). PE lessons and break times accounted for 15 ± 13 min (12.7 %) and 7 ± 6 min (5.8 %) of daily MVPA, respectively. On days with PE, children spent 144 ± 68 min in MVPA, whereas on days without PE, this time decreased significantly to 122 ± 63 min (*p* ≤ 0.01).

**Conclusion:**

The findings suggest that segments such as PE lessons and morning breaks are important sources for MVPA for boys and girls. This should therefore be considered for policies, timetables and curriculums in order to offer sufficient opportunities for children to be physically active during the school day.

## Background

The prevalence of childhood overweight and obesity has increased considerably during the past 30–40 years, and thus is of public health concern (Ogden et al. 2010). Data from the US, Australia and many European countries show a consistent rise in BMI and body fat in children (Ng et al. 2014; Pigeot et al. 2010), which is considered to have extensive health implications including early type 2 diabetes, hyperinsulinemia and cardiovascular disease (Després et al. 2008). Although, a plateauing of childhood obesity has recently been suggested (Wabitsch et al. 2014), numbers from Germany show an increase of 150 % for body weight when comparing children’s data from the 1970s to those from 2006 (Nagel et al. 2009). Subcutaneous fat in 6–9 year old children has been shown to increase even three to fivefold (Nagel et al. 2009), which means, even if levels have plateaued, children’s weight status stopped at a very high level. An extensive body of research indicates that overweight and obese children have a higher risk of becoming obese adults than their normal counterparts (Singh et al. 2008; Venn et al. 2007; Pei et al. 2013; Wheaton et al. 2015) and a major contributing factor is low levels of physical activity (Hills et al. 2011). On the other hand, benefits of a physically active lifestyle for childhood health are well documented (Andersen et al. 2006; Janssen and LeBlanc 2010); therefore, the WHO (WHO 2010) developed recommendations for appropriate amounts of physical activity for children and adolescents. In spite of this, many children are not sufficiently active enough to benefit their health. Substantial evidence documents that nearly 50 % of youth in the US fail to meet the minimum physical activity guidelines (Song et al. 2013) and only 32 % of boys and 24 % of girls in England aged 2–15 years meet recommendations of 60 min or more of moderate to vigorous physical activity (MVPA) a day (US Dept. Health Services 2008). German data shows that not even 20 % of children between the ages of 7 and 10 years are sufficiently active to meet the WHO guideline (Krug et al. 2012). These differences may at least partially be due to variations in assessing children’s physical activity which has been shown to be influenced by different interpretations, cut-off points and measurement durations (Guinhouya et al. 2013). These findings, however, suggest the need for a more detailed understanding into children’s physical activity patterns to more effectively tailor physical activity promotion strategies in this population. Because the majority of youth can be reached at school, interventions delivered there are appealing; so far, however, detailed and objective insight of children’s physical activity levels throughout the school day is lacking.

During the school morning, break times and physical education (PE) lessons are often the only opportunities for children to accumulate time spent in physical activity. Nonetheless, at schools allocating sufficient time for PE and other physical activity opportunities during the school day has become increasingly difficult, mainly due to budgetary restrictions and decisions to support other academic areas (McMurrer 2008; Wilkins et al. 2003). As a result, PE lessons and activity breaks are often eliminated or reduced (McMurrer 2008; Amis et al. 2012).

Given the fact, that the degree of correlation between physical activity and some health benefits are intensity driven (US Dept. Health Services 2011), it is important to gain an understanding of which parts of the day may promote an increased engagement in MVPA. There are some published studies of objectively determined physical activity during the school day in primary school children (Bailey et al. 2012; Fairclough et al. 2007, 2012; Tudor-Locke et al. 2006); however, hardly any of those are based in Germany, which has a different schooling system to most other European countries, the US and UK. Generally, most German primary schools finish at lunchtime with children spending no more than 5 h daily at school and, therefore, missing the opportunity for a lunchtime activity break at school, which makes previous research only partially applicable. The aim of this study, therefore, is to objectively investigate the amount and intensity of German primary school children’s physical activity during different segments of the school day and to explore the percentage of PE lessons and break times spent in daily MVPA.

## Methods

### Participants

A total of 294 primary school children (7.1 ± 0.7 years; 48 % male), a sub-sample of 1947 children participating in the school-based health-promotion programme “Join the Healthy Boat” (Dreyhaupt et al. 2012; Kobel et al. 2014) in south-west Germany, were used for analysis. All data are baseline measurements, prior to any intervention. The sub-sample, who agreed to take part in objective physical activity measurements, did not differ from the rest of the children taking part with regards to gender, age, anthropometry (incl. weight status), sports participation, migration status and parental education level. Parents’ written informed consent as well as child assent were obtained prior to data collection. The study was approved by the Ministry of Culture and Education as well as the University’s ethics committee and is in accordance with the declaration of Helsinki.

### Anthropometric measures

During a school visit, children’s height (cm) and body mass (kg) were taken by trained staff according to ISAK procedures (Stewart et al. 2011). Height and weight were measured to the nearest 0.1 cm and 0.05 kg, respectively (Seca 217 and 826, respectively; Seca Weighing and Measuring Systems, Hamburg, Germany). Using German reference data (Kromeyer-Hauschild et al. 2001), children’s body mass index (BMI) was converted to BMI percentiles (BMIPCT). Weight status was subsequently classified as normal weight (until 90th percentile) and overweight/obesity (above 90th/97th percentile).

### Physical activity

To assess physical activity, the children wore a multi-sensor device (Actiheart, CamNtech Ltd., Cambridge, UK), which is attached to the child’s chest and measures bodily movement simultaneously with heart rate (Brage et al. 2005). The Actiheart has been validated previously and shown to reliably predict energy expenditure during common activities in free-living situations in children (Corder et al. 2007). Energy expenditure in METs was derived from Actiheart’s captive software (version 4.0.73), utilising participant’s age, height, body weight and gender in addition to the recorded heart rate and movement counts to assess physical activity intensity. Activity levels were then classified as sedentary (< 1.5 MET), light (1.5–3 MET), moderate (3–6 MET), and vigorous (> 6 MET) (Pate et al. 1995).

With recording intervals set to 15 s, participants wore the multi-sensor device for 6 days consecutively (à 24 h). To be included in the analysis, at least 3 days with valid data of more than 10 h daily were required. To antagonise novelty, the first day was excluded from analysis, as was the last recording day, which never showed 10 h of recording. In order for children to be classed as meeting current physical activity guidelines, 60 min of MVPA were required on each day of physical activity recording.

### Physical education and break times

Individual PE lessons and break times during school mornings were identified for each child using timetables provided by the participant’s teachers. Activity intensities and time spent in those during PE lessons and break times were individually calculated. Weekdays were subsequently allocated to “PE days” (with at least one PE lesson scheduled) and “non-PE days” (days without PE lessons).

### Statistics

Data were initially checked for compliance to the monitoring protocol (i.e. valid data for at least 10 h daily on at least 3 days) resulting in 294 children with valid activity data and information about break times and PE lessons. Descriptive statistics were calculated (mean values and standard deviations, SD). All statistics were performed using SPSS Statistics 19 (SPSS Inc., Chicago, IL, US) using a significance level of *p* ≤ 0.05. After non-normal distributions of the data have been shown using Kolmogorov-Smirnov-Tests, Mann-Whitney-U-Tests were used to examine group differences. Logistic regressions were used to determine odds ratios (OR, with 95 % confidence interval) of meeting the physical activity guideline or not.

## Results

Table 1 shows a summary of the participants’ anthropometric characteristics. No significant gender differences were found.On a daily average, children spent 132.7 (± 61.1) minutes in MVPA; when only considering weekdays; however, this amount increased significantly (140.7 ± 65.8 min, *p* ≤ 0.01). As shown in Table 2, boys spent significantly more time in MVPA than girls throughout the whole assessment period as well as on weekdays (*p* ≤ 0.01). Thus, 45.9 % of children reached the recommended activity guidelines of 60 min of MVPA daily, again with boys achieving this goal significantly more often than girls—65.6 vs. 28.7 %, respectively; *p* ≤ 0.01; OR 0.19 (0.111; 0.325). Weight status also affected reaching 60 min of MVPA daily. Overweight and obese children reached guidelines significantly more often than normal weight children—72 vs. 43 %, respectively, *p* ≤ 0.01; OR 4.39 [(.66; 11.63).

**Table 1:** Participant’s characteristics

	Boys	Girls	All
*N* (*n*; %)	140 (47.6)	154 (52.4)	294 (100)
Age (years)	7.2 (0.7)	7.1 (0.7)	7.1 (0.7)
Height (cm)	124.2 (6.5)	123.2 (6.3)	123.7 (6.4)
Body Mass (kg)	25.1 (5.3)	24.4 (4.9)	24.7 (5.1)
BMIPCT	49.7 (26.9)	47.7 (29.0)	48.6 (28.0)
Overweight/obese (%)	5.7/4.3	5.2/4.6	5.5/4.4

Values are displayed in mean and SD. *BMIPCT* body mass index percentiles

**Table 2:** Moderate to vigorous physical activity (MVPA) throughout the segmented day

	Boys	Girls	All
MVPA_total_ (min)*	160.9 (58.6)	107.8 (51.9)	132.7 (61.1)
MVPA_weekdays_ (min)*	169.9 (66.3)	115.0 (53.7)	140.7 (65.8)
Reached Guideline (%)*	65.6	28.7	45.9
MVPA_break time_ (min)*	8.4 (6.2)	5.3 (4.7)	6.8 (5.7)
MVPA_PE_ (min)*	17.0 (13.9)	12.7 (11.0)	14.8 (12.7)
MVPA_break time_ (%_of total_)	5.8 (4.0)	5.8 (5.6)	5.8 (4.9)
MVPA_PE_ (%_of total_)	11.3 (8.6)	13.9 (13.2)	12.7 (11.3)
MVPA_PE day_ (min)*	172.2 (70.2)	119.7 (55.6)	144.4 (68.0)
MVPA_non-PE day_ (min)*	150.5 (57.4)	97.4 (57.6)	122.2 (63.3)

MVPA during the assessment period, weekdays, break times, physical education (PE) classes, days with PE and days without PE, split by gender

Values are displayed in mean and SD in minutes (min) or percentages (%)

* Significant gender differences (*p* ≤ 0.05)

All children participated in 135 min PE per week (three lessons at 45 min each) and had break times varying between 15 and 90 min per school morning with an average of 30.7 (± 13.8) minutes. PE lessons and morning break times accounted for 14.8 (± 12.7) minutes and 6.8 (± 5.7) minutes of MVPA, respectively. Equating to 12.7 and 5.8 % of total daily MVPA, respectively. Boys spent significantly more time in MVPA during both, PE lessons and break times (*p* ≤ 0.01). In girls, MVPA spent during PE lessons accounted for a higher percentage of their daily MVPA than in boys; this however was not significant (*p* ≤ 0.07). Similarly, in normal weight children, MVPA accumulated during break times accounted for a higher percentage of their daily MVPA than in overweight and obese children (*p* ≤ 0.03).

**Fig. 1:**
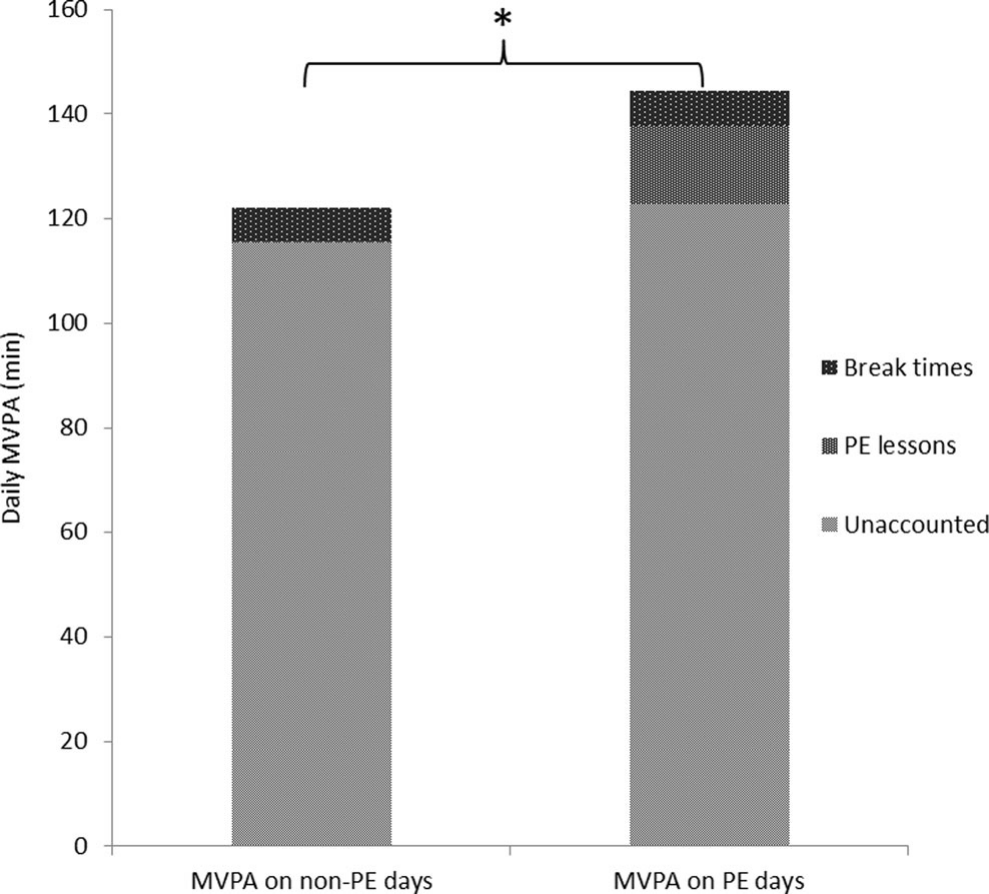
Moderate to vigorous physical activity (*MVPA*) on days with and without physical education (*PE*). Average daily MVPA in minutes (min) on days with PE lessons and days without PE lessons, split into MVPA during break times, PE lessons and unaccounted MVPA during the assessment period. The asterisk refers to significant difference in total MVPA (*p* ≤ 0.05)

Physical activity during break times and PE lessons therefore accounted for 18.5 % of children’s daily MVPA (17.1 % for boys and 19.7 % for girls). On days with PE lessons, children spent 144.4 (± 68.0) minutes being moderately to vigorously active, whereas during days without scheduled PE lesson, this time decreased significantly to 122.2 (± 63.3) minutes (*p* ≤ 0.01; see Fig. 1). However, when deducting time spent in MVPA during PE lessons off total MVPA on school days with PE lessons and comparing it with MVPA on non-PE days, there is a difference of 7.2 (± 61.1) minutes, thus not significant because of the large range (−381.2 to 246.0).

A further gender difference could be observed, with boys spending significantly more time in MVPA during PE days and non-PE days (*p* ≤ 0.01; Tab. 2). Comparing normal weight with overweight and obese children, the latter spent significantly more time in MVPA during PE days (141.1 ± 67.9 vs. 170.9 ± 60.5 min, respectively; *p* ≤ 0.04) as well as during non-PE days (117.3 ± 62.4 vs. 164.7 ± 55.1 min, respectively; *p* ≤ 0.01).

## Discussion

The results of this study extend the current literature by providing a detailed analysis of children’s time spent in MVPA during specific segments of the school day. Current physical activity guidelines recommend children should engage in at least 60 min of MVPA daily (WHO 2010). The results of this study suggest that segments such as PE lessons and morning breaks are important sources for MVPA engagement in boys and girls; especially when considering that children’s overall physical activity levels were higher during weekdays. The latter may at least partly be due to parenting practices but also to schools offering children an environment with structured (physical education) and unstructured (break times) occasions for physical activity accompanied by their peers (Fairclough et al. 2012). In the present study both aspects were investigated and assessed objectively. Current recommendations for primary schools are to provide children with at least 30 min of MVPA daily during PE, break times and other opportunities (Centers of Disease Control and Prevention 2011). Overall a minimum of 20 min of morning break times and 150 min or more per week during PE lessons with at least 50 % of MVPA during PE time are suggested (Centers of Disease Control and Prevention 2011; Koplan et al. 2005; Pate et al. 2006). In this study, children received 135 min of PE per week and averaged just over 30 min of break times daily.

Children’s PE lessons accounted for a total of 14.8 min of MVPA equating to virtually 13 % of their total daily MVPA. Although boys spent significantly more time in MVPA during PE lessons, in girls, MVPA spent during PE lessons accounted for a higher percentage of their daily MVPA than in boys; this however was not significant. Similar values have been shown by US research assessing 11 year old children during PE classes using pedometers, resulting in the same number of steps in boys and girls, but PE accounting for 8 and 11 % of total daily physical activity for boys and girls, respectively (Tudor-Locke et al. 2006).

Further, boys’ morning break times accounted for 8.4 min of MVPA, whereas girls engaged in 5.3 min of MVPA during morning break, equating to almost 6 % of total daily MVPA in boys and girls. Although boys spent significantly more time in MVPA during both, PE lessons and break times, their physical activity during these times only accounts for 17 % of their daily MVPA. This is considerably lower than findings by UK and US research, where in-school physical activity accounted for 30–37 % of children’s total MVPA (Gidlow et al. 2008; Brusseau et al. 2001). Again, this discrepancy may be due to the different schooling systems, since children in the UK spend noticeably more time in school than do German children, who finish school at lunchtime. It was also noted that for boys lunchtime physical activity provided the largest amount of physical activity at school, followed by PE and break times (Brusseau et al. 2001) which confirms the aforementioned assumption. For girls on the other hand, PE was suggested to provide the largest amount of physical activity at school (Brusseau et al. 2001).

This study also shows that PE has a positive effect on children’s school day physical activity levels. On days with PE lessons, children spent 144 min being moderately to vigorously active, whereas during days without scheduled PE lesson, this time decreased significantly to 122 min. This is in accordance with previous literature, showing PE to be a major source of physical activity during school days (Bassett et al. 2013). However, when deducting time spent in MVPA during PE lessons off total MVPA on school days with PE lessons and comparing it with MVPA on non-PE days, there is a difference of 7.2 (±61.1) minutes; thus, it is not significant because of the large range. This indicates that children are more active on PE-days but not just during the PE lessons, but also during the rest of the day. Due to the chosen measurement method, it remains unclear why children are more active on days that offer more physical activity at school, but very early research has shown that children do not seem to compensate for a sedentary school day by being more physically active after school (Dale et al. 2000). Quite the contrary, activity levels after school were shown to be higher after an active school day (Dale et al. 2000).

Nonetheless, children in this study accumulated on average more than 20 min of MVPA during break times and PE lessons, which is two-thirds of recommended guidelines of ≥ 30 min/day of MVPA during school (Centers of Disease Control and Prevention 2011; Koplan et al. 2005; Pate et al. 2006). Structured and unstructured offer children the option to be engaged in more physical activity at school, whether during break times, in PE classes or in classroom activity breaks, and could enable children to attain more easily and more frequently the 30 min of MVPA at school in order to reach the recommended daily amount of 60 min of MVPA (WHO 2010) . Furthermore, recent research has shown that providing various activity opportunities at school (such as sufficient PE or supervised break times) doubles children’s physical activity during school (Carlson et al. 2013). Together, these data suggest that encouraging physical activity as part of the school day may also promote higher levels of physical activity after school.

Moreover, and in line with previous research, this study showed a gender difference with boys spending significantly more time in MVPA irrespective of PE days or non-PE days. This gender difference is well reported in school day physical activity as well as general physical activity (Stratton et al. 2007; Tudor-Locke et al. 2006; Fairclough et al. 2012). It seems when there are possibilities for physical activity, boys engage themselves with higher intensity than girls and have a preference for outdoor play (Ridgers et al. 2006; Trost et al. 2002), whereas girls engage in more MVPA if there is a nicely designed playground (Möhrle et al. 2015). However, although boys were still more active during PE classes compared to girls, an increase in MVPA could be observed for girls and boys, which indicates that both, boys and girls, benefit from having PE at school.

Not only gender, but also weight status showed to be connected to time spent in MVPA, which is in alignment with previous studies (Ruiz et al. 2006; Dencker et al. 2008). Comparing normal weight with overweight and obese children, this study showed that the latter spent significantly more time in MVPA throughout the school day, irrespective of PE, where weight status showed no difference. On the other hand, in normal weight children, MVPA accumulated during break times accounted for a significantly higher percentage of their daily MVPA than in overweight and obese children, which supports early studies on physical activity during break time in primary school children (Stratton and Leonard 2002; Ridgers et al. 2006). These differences have previously been attributed to discrepancies in fundamental movement skills, which differ between normal weight and overweight children (Okely et al. 2004).

Nevertheless, there are limitations, which should be considered when interpreting these data. First, the cross-sectional data does not allow for causality. Although observing children’s activity levels for 6 days, including three to four school mornings, direct observation may have enabled more thorough and more in-depth results. For example, no quantitative information about the time children spent being instructed, getting changed and being active was provided by the teachers and neither heart rate nor accelerometry provide any contextual information about the monitored lessons. Also, it is possible the PE lessons were taught differently, and children acted differently as a result of some children being monitored by multi-sensor devices. Moreover, weather might have affected children’s activities during their morning breaks and therefore influenced their physical activity levels. Additionally, while the study’s schools are spread throughout the south-west of Germany including urban and rural settings, the findings are not generalizable, especially because of the low prevalence of overweight and obese children in this sample. In order to answer more contextual questions about children’s physical activity behaviours, future work should combine objective measures with direct observation.

## Conclusion

The world-wide increase in obesity in children together with their low physical activity levels give reasons to search for effective ways to increase children’s MVPA and, therefore, potentially reduce overweight and obesity in children. The results of this study extend the current literature by providing a detailed analysis of children’s time spent in MVPA during specific segments of the school day. Current physical activity guidelines recommend children should engage in at least 60 min of MVPA daily. This study’s results suggest that schools offer an optimal environment to increase children’s physical activity levels; especially segments such as PE lessons and morning breaks are important sources for MVPA engagement in boys and girls. This should, therefore, be considered for policies, timetables and curriculums in order to offer sufficient opportunities for children to be physically active during their school day.

## Acronyms

BMI: Body mass index
BMIPCT: Body mass index percentiles
MVPA: Moderate to vigorous physical activity
PA: physical activity
PE: Physical education
SD: Standard deviation
US: United States of America
WHO: World Health Organisation
